# Replacing Soybean Meal with Urea in Diets for Heavy Fattening Lambs: Effects on Growth, Metabolic Profile and Meat Quality

**DOI:** 10.3390/ani9110974

**Published:** 2019-11-14

**Authors:** Cristina Saro, Javier Mateo, Sonia Andrés, Iván Mateos, María José Ranilla, Secundino López, Alba Martín, Francisco Javier Giráldez

**Affiliations:** 1 Instituto de Ganadería de Montaña, CSIC-Universidad de León, Finca Marzanas s/n, Grulleros, 24346 León, Spain; sonia.andres@eae.csic.es (S.A.); imata@unileon.es (I.M.); mjrang@unileon.es (M.J.R.); s.lopez@unileon.es (S.L.);; 2 Departamento de Higiene y Tecnología de los Alimentos, Universidad de León, Campus Vegazana, s/n, 24071 León, Spain; jmato@unileon.es; 3 Departamento de Producción Animal, Universidad de León, Campus Vegazana, s/n, 24071 León, Spain; amartg18@estudiantes.unileon.es

**Keywords:** Assaf lambs, urea, feed efficiency, rumen fermentation, meat quality, metabolic profile, acidosis

## Abstract

**Simple Summary:**

There is a renewed interest on the potential inclusion of urea in ruminant diets, reducing the contribution of vegetable protein supplements. This study was designed to evaluate the effect of replacing soybean meal with different proportions of urea in protein-rich diets for heavy fattening lambs (from 29 to 50 kg of live body weight). Our results suggest that 39% of soybean meal of such diets can be replaced with urea reducing the feeding costs without any adverse effects on feed efficiency, rumen fermentation, or carcass and meat quality. Nevertheless, urea supplementation even at levels of 1% of dry matter may trigger mild metabolic acidosis that can affect animal health in the long term.

**Abstract:**

Thirty-six Assaf male lambs (29.4 ± 3.10 kg body weight (BW)) were used to study the feasibility of including urea (at 0, 0.6 or 0.95% of dry matter for Control, Urea1, and Urea2 diets, respectively) in substitution of soybean meal in fattening diets. Animals were individually penned and feed intake was recorded daily. Blood samples were taken at days 35 and 63 of the experimental period to determine the acid-base status and the biochemical profile. At the end of the experiment (nine weeks), lambs were slaughtered, ruminal contents were collected and carcass and meat quality were evaluated. There were not differences (*p* > 0.05) among treatments in dry matter intake, animal performance, ruminal fermentation pattern, and carcass and meat parameters. Serum albumin concentration was higher and concentration of HCO_3_ and total CO_2_ in blood were lower in Urea2 compared to Urea1 and Control lambs. These results, together with the tendency to lower (*p* = 0.065) blood pH in this group might suggest a moderate metabolic acidosis. Partial replacement of soybean meal with urea did not impair growth rate in heavy fattening Assaf lambs (from 29 to 50 kg body weight), reduced feeding costs and had no adverse effects on feed efficiency, rumen fermentation and carcass and meat quality.

## 1. Introduction

Global demand of vegetable protein feeds for livestock is growing as a result of the increased demand for animal-based protein. It is expected that livestock production by 2020 will become the most important agricultural sector in terms of added value, if the challenges related to environmental impact and competition for resources are adequately addressed [1,2,3,4]. In this context, the European Union is increasingly aware of the deficit in vegetable protein for animal feeding and alerts about the importance to reduce the massive dependency on imports of protein crops because of the environmental impact in producing regions and the volatility of the protein prices in international markets [5].

Research on urea utilization in ruminant feeding was promoted in the interwar period as a consequence of the critical shortage of vegetable protein supplements for livestock. Thus, from 1950s urea became a generally accepted ingredient in ruminant diets [6]. Current situation of the growing global demand of vegetable protein for livestock feeding has renewed interest in extending the use of urea as much as possible in ruminant feeding, and a research effort is being made to establish the optimal level of this non-protein nitrogen source for each specific nutritional situation [6,7,8].

Classical studies have demonstrated that growing-fattening lambs do not use urea as efficiently as mature sheep or cattle and that the benefits of replacing conventional protein supplements with urea depend mainly of the dietary protein content [9,10]. Regarding to this, it has been corroborated that urea is an adequate source of N in diets not exceeding 12–13% of crude protein (CP) and conventional protein feed supplies at least the 25% of total CP [11]. However, unsatisfactory results have been reported when more than 25% of the protein is replaced with urea in diets with higher CP content [10,12]. Different studies suggest that dietary CP content in diets for growing Assaf lambs, at least up to 50 kg of body weight (BW), should be greater than 13.6% in order to achieve an average daily gains (ADG) higher than 300 g/day [13,14]. Under these conditions, the maximal inclusion level of urea is not well established.

On the other hand, most of the studies investigating the use of urea in growing and fattening lambs have focused on its effects on animal health and zootechnical performance, with little available information regarding the effects on meat quality. However, recently it has been reported that urea supplementation can affect relevant meat quality parameters such as tenderness, lightness, and redness, although these effects seem to be dose-dependent and could be affected also by dietary CP content and growing phase [15,16].

The objective of the present study was to assess the effects of two different levels of inclusion of urea replacing partially soybean meal in high protein diets for heavy fattening lambs (from 29 to 50 kg BW) on animal performance, ruminal fermentation, blood acid-base status, biochemical profile, and carcass and meat quality.

## 2. Materials and Methods

All handling practices followed the recommendations of the Directive 2010/63/EU of the European Parliament and of the Council on the protection of animals used for scientific purposes and the CSIC Animal Experimentation Committee (protocol number 100102/2017-4) authorized all the procedures included in the experimental design.

### 2.1. Animals and Diets

Thirty-six male Assaf lambs (29.3 ± 3.10 kg BW and 81 ± 0.8 days of age) were used. Animals were randomly allocated to three experimental groups (12 animals per treatment group, equilibrated by BW and age) and fed the following diets: Control (with no urea), Urea1 (6 g of urea/kg dry matter (DM)), and Urea2 (9.5 g of urea/kg DM). The non-protein N supplement used in diet formulation was feed-grade urea (Urea Rumisan, Yara International). Ingredients and chemical composition of the diets are shown in Table 1. They were formulated to be isoenergetic and isonitrogenous, with urea replacing 25% (Urea1) or 39% (Urea2) of the soybean meal in the control diet.

### 2.2. Experimental Procedures

Animals were individually penned during the whole experimental period (nine weeks). Lambs were offered ad libitum the experimental diets once a day at 09:00 h and refusals were recorded daily and composited weekly to determine DM content. The amount of feed offered was readjusted daily in order to allow 10% refusals and fresh water was always available. Diets were pelleted to avoid feed selection by lambs.

All lambs were weighed once a week throughout the experimental period. On days 0, 35 and 63 blood samples were taken before morning feeding by jugular venipuncture into tubes containing lithium heparin. Tubes were placed in ice and then centrifuged (3520× *g* for 20 min at 4 °C) and plasma samples were frozen at −20 °C until analysis of biochemical profile. On days 35 and 63 another blood sample was obtained for acid-base status evaluation.

The last day of the experiment, lambs were transported to a commercial abattoir 2 h before slaughtering. The transfer of the animals from the research facilities to the abattoir lasted approximately 15 min (without intermediate stops), and the handling of animals during transportation and slaughtering followed strictly the Council Regulation (EC) No. 1099/2009 on the protection of animals at the time of killing. Feed had been withdrawn, and after an 8-h fasting, lambs were weighed, stunned, slaughtered by exsanguination from the jugular vein, eviscerated and skinned. In four representative lambs of each group, the rumen was dissected and the whole ruminal contents were mixed and strained through four layers of cheesecloth. The pH of the filtered fluid was measured immediately. Then, a 1 mL sample was added to 1 mL of 0.5 N HCl for ammonia-N determination, and a sample of 0.8 mL was added to 0.5 mL of deproteinizing solution (20 g metaphosphoric acid and 0.6 g crotonic acid/L in 0.5 N HCl) for volatile fatty acids (VFA) analysis.

After sampling, the strained rumen fluid was used for an in vitro assay to assess rumen fermentation by incubating the experimental diet that the animal had received during the trial. Rumen fluid was mixed with a buffered culture medium [17] in a 1:4 proportion. This medium had been extensively reduced with continuous bubbling of CO_2_ and warmed to 39 °C. Incubations were performed in 120 mL serum bottles in which 300 mg of DM substrate (experimental diet) had been weighed. Rumen fluid from each animal was used as a separate inoculum, thus having four replicates per treatment.

Two bottles per animal and blanks were included in the incubation trial. After dispensing anaerobically 30 mL of diluted rumen fluid, each bottle was sealed with rubber stoppers and aluminum seals and all the bottles were placed in an incubator at 39 °C for 24 h. At the end of the incubation total gas production was determined following the method proposed by Theodorou et al. [18] by using a pressure transducer (Delta Ohm DTP704-2BGI, Herter Instrument SL, Barcelona, Spain) and a calibrated syringe. The syringe plunger was withdrawn until the gas pressure in the head-space of the bottles was returned to ambient pressure, as indicated by a reading of zero on the display of the transducer, and a gas sample (10 mL) was taken into a 10 mL vacuum tube (Venoject, Terumo Europe N.V., Leuven, Belgium) for methane analysis. Bottles were swirled in ice to stop fermentation, and then opened to measure pH in the incubation medium and samples for ammonia·-N and VFA analysis were taken as previously described.

DM content of feeds and refusals were determined according to ISO 6496:1999 [19]. Ash and CP in feed samples were analyzed following the ISO 5984:2002 [20] and ISO 5983:2009 [21] procedures, respectively. Neutral-detergent fiber (NDF) and acid-detergent fiber (ADF) were analyzed as described by Van Soest et al. [22] using an Ankom fiber analyzer (Ankom Technology Corp., Macedon, NY, USA).

Frozen plasma samples were defrosted overnight at 4 °C and aspartate aminotransferase (AST), alanine aminotransferase (ALT), total cholesterol, glucose, triglycerides, calcium, phosphorus, urea, creatinine, total proteins, and albumin were determined in a biochemical profile autoanalyzer ILAB 650 (Instrumentation Laboratory, Lexington, MA, USA). Blood acid-base status was evaluated in fresh blood samples by determining pH, bicarbonate (HCO_3_−), CO_2_ pressure (pCO_2_), anion gap, total CO_2_ (tCO_2_), Na, K, and Cl concentrations in a VetStat blood gas and electrolytes analyzer (Idexx, Barcelona, Spain).

Concentrations of ammonia-N and VFA were determined as described by García-Martínez et al. [23] using a GC-2010 gas chromatograph (Shimadzu, Duisburg, Germany) and CH_4_ was analyzed by gas chromatography as described by Martínez et al. [24].

Average daily gain (g/day) was estimated as the regression coefficient (slope) of body weight against time. The feed conversion ratio was obtained dividing the average daily DMI by the estimated ADG. Residual feed intake (RFI), an index of feed efficiency, was calculated as described by Koch et al. [25]. Briefly, estimated feed intake was calculated as the regression of the real feed intake against mid-test metabolic weight and average daily gain. The difference between the actual and the estimated feed intake represents the RFI.

### 2.3. Carcass and Meat Characteristics

Carcass weight was recorded before (HCW) and after chilling (CCW) at 4 °C for 24 h and refrigeration losses were calculated. Dressing percentage was calculated as a proportion of the CCW on slaughter weight.

The color parameters [L* (lightness), a* (redness), and b* (yellowness) (D65 illuminant, 10° visual angle, SCI mode, 11 mm aperture for illumination and 8 mm for measurement)] of subcutaneous fat were measured in the lumbar region with a Minolta CM-2002 chroma meter (Konica-Minolta Sensing, Inc., Germany) using the CIELAB system [26]. The hue angle (h*), which indicates a combination of red and yellow (0° is red; 90° is yellow), was calculated as arc tangent (b*/a*), and the chroma (C*), an index of purity of color that describes the vividness or dullness of a color (0 is dull; 60 is vivid), was computed as $C=\sqrt{a^{2}+b^{2}}$. The pH value from the *Longissimus thoracis* muscle (left side) was measured 24 h postmortem at the sixth rib using a Metrohm^®^ pH meter equipped with a penetrating electrode.

Carcasses were halved carefully and the left side was then divided into commercial cuts as described by Colomer-Rocher et al. [27] and weighed. Legs, loin, and fore ribs comprised the higher priced joints, shoulders were the medium price joints, and the lower priced joints included breast, neck, and tail. Dissection of the leg was performed using the method of Fisher and de Boer [28] to determine the tissue composition.

The *Longissimus thoracis* (LT) and *lumborum* (LL) muscles were removed from the ribs. Four slices from the distal end of the *L. thoracis*, each 2.5 cm thick, were cut and in two sets of two slices per set were placed on impermeable polypropylene trays, which were wrapped with polyvinylchloride cling film and refrigerated stored (4 °C) in darkness. The color was determined after one hour of cutting (day one; approx. 26 h after slaughter) on the surface of the two slices of one of the sets and on those of the other set after six days of storage (day six). Color was determined in duplicate on each of the slices following the procedure previously described. The remaining portion of *L. thoracis* muscle, i.e., the portion of muscle not used for color determination, was trimmed to eliminate connective tissue, minced in a food processor, and frozen at −20 °C until analysis. Chemical composition was determined in accordance with the methods described by the Association of Official Analytical Chemist [29].

The LL muscle was used to determine cooking losses and hardness (shear force). The muscle from each carcass was transversely divided in two similar portions (distal and caudal), which were randomly allotted to two different measurement times (day one or day six). The muscle portion for day one was immediately weighed, vacuum packaged, and cooked in a water bath at 80 °C for 40 min, cooled with tap water, removed from the packaging bag, dried on its surface with filter paper and weighed again. Cooking loss was then determined as the difference in weight between cooked and raw meat, expressed as percentage of raw meat. The other portion was refrigerated stored for six days under the same conditions as described for the *L. thoracis* (color determination) before being vacuum packaged, cooked, cooled, and weighed. Afterwards, three cuboid core samples (square cross-section (1 cm by 1 cm) and with the long axis (3 cm) parallel to the predominant muscle fiber orientation) were obtained from the cooked LL muscle portions (days one and six) and subjected to Warner–Bratzler shear force analysis following the procedure described by Santos et al. [30].

### 2.4. Calculations and Statistical Analysis

Data from growth performance, feeding costs, carcass characteristics, in vivo rumen parameters, tissue and meat chemical composition were analyzed using the MIXED procedure of SAS (SAS Inst. Inc., Cary, NC, USA), including the fixed effect of the diet in the model and animal nested within the diet as residual error. Initial BW was also included as a covariate in the model but it was removed as the effect was non-significant (*p* > 0.05). In vitro data (average of the two values per animal) were analyzed using a mixed model with the fixed effect of the diet and the animal nested within the diet as random effect.

Data from blood acid-base status and biochemical parameters, texture, cooking losses, and color of the meat were analyzed as a repeated measures model using the MIXED procedure of SAS. The animal was nested within the diet and considered the experimental unit to test the effect of diet. Different covariance matrices were evaluated on the basis of Schwarz’s Bayesian information criteria. Plasma values at day zero were used as covariate, the covariate being removed from the model when its effect was not significant (*p* > 0.05). The effect of the covariate was significant (*p* < 0.05) for glucose, albumin, urea, AST, creatinine, Ca, and P concentrations and hence adjusted mean values were used.

## 3. Results

### 3.1. Feed Intake and Animal Performance

Dry matter intake, daily body weight gain, feed to gain ratio, residual feed intake, and final BW for each experimental diet are shown in Table 2. There were not differences (*p* > 0.05) for any of the parameters recorded.

### 3.2. Ruminal Parameters, Blood Acid-Base Status and Biochemical Profile

No difference (*p* > 0.05) was observed in any of the fermentation parameters recorded in the rumen fluid samples taken at slaughter among the three groups (see Table 3). In the in vitro assay, VFA production was reduced in the Urea1 group compared with Control and Urea2 groups, with no differences in the other parameters evaluated.

Blood parameters related to acid-base status corresponding to the different experimental treatments and sampling days are shown in Table 4. As the effect of interaction between diet and day was not significant for any of the parameters evaluated, only main effects are shown.

HCO_3_^−^ and tCO_2_ were lower (*p* ≤ 0.011) in Urea2 than in Control lambs, while Urea1 group showed intermediate results. Likewise, blood pH in Urea2 lambs showed a tendency (*p* < 0.065) to be lower than in Control ones. The other parameters were not affected by dietary treatments. HCO_3_^−^, tCO_2_, Anion gap, pH and K and Cl values were affected (*p* ≤ 0.017) by sampling day. Anion gap was highest on day 63, and the rest of the parameters showed lowest values in the last day of measure. Concentration of Na and pCO_2_ were unaffected (*p* < 0.05) by sampling time.

The effect of urea supplementation and day upon biochemical profile is presented in Table 5. Increasing levels of dietary urea had no effect (*p* > 0.05) on ALT, AST, protein, urea, glucose, cholesterol, triglycerides, and Ca concentrations. However, albumin, creatinine, and P concentration were affected by dietary treatments (*p* ≤ 0.027), so that mean values for Control lambs were lower than those showed by lambs fed containing urea diets. Plasma concentration of urea, protein, creatinine, P, cholesterol, and triglycerides were also affected (*p* ≤ 0.013) by sampling day. Urea, creatinine and cholesterol concentrations increased from day 35 to 63, whereas protein, triglycerides and *p* values decreased. Diet × day interaction effect was only significant (*p* = 0.013) for Ca, as Ca was higher in day 35 than in day 63 but only in control (12.0 versus 11.1 mg/dL; means within the treatment) and Urea2 lambs (12.7 versus 11.4 mg/dL; means within the treatment).

### 3.3. Carcass and Meat Characteristics

As shown in Table 6, the effects of the diet on carcass performance were not significant (*p* > 0.05).

Meat quality data are presented in Table 7. No differences (*p* > 0.05) were observed among experimental groups in chemical composition, cooking losses, texture, and color parameters. Shearing force decreased and redness, yellowness and chroma values increased during storage, from day one to day six after slaughtering.

### 3.4. Feeding Costs

There were not differences among treatments (*p* = 0.117) in total feeding cost (€/lamb) during the fattening period (from 29 to 50 kg of BW) among treatments, with mean costs of 37.1, 33.3, and 34.7 €/lamb for control, Urea1, and Urea2 groups, respectively. Nevertheless, differences among treatments (*p* < 0.05) were found when feeding cost was expressed in relation to either ADG (1.90, 1.72, and 1.71 €/kg ADG for Control, Urea1, and Urea2, respectively) or CCW (1.49, 1.39, and 1.41 €/kg CCW for control, Urea1, and Urea2, respectively).

## 4. Discussion

### 4.1. Feed Intake and Animal Performance

The major adverse effects observed when urea has been used in ruminant feeding are feed intake depression and toxicity, which are related to several factors such as total daily ingestion of urea, dietary CP and fermentable energy contents, and proportion of the CP supplied as non-nitrogen protein [31]. In order to prevent these adverse effects it is recommended to use urea in starch-rich diets, not to include more than 1% of urea and not to substitute more than one third of the CP with non-protein nitrogen, although controversial results have been reported [31,32]. In the current trial neither feed intake depression nor signs of toxicity were observed. Moreover, dry matter intake was similar to that reported by Abo Omar and Nasser [33] in male Assaf lambs at a similar BW range and receiving medium (14%) and high (18%) protein diets without urea supplement.

In agreement with the lack of effect on feed intake, average daily gain, feed conversion rate, or residual feed intake were not affected by urea supplementation, with mean values similar to those reported for Assaf lambs fed high concentrate diets [33,34]. In contrast to our results, several studies reported negative effects of urea supplementation on daily gain and feed to gain ratio in growing ruminants, with or without a simultaneous effect on feed intake with similar or higher levels of inclusion to those used in this study [15,35,36]. These controversial results as suggested by Zinn et al. [35] could be related with the use of low energy finishing diets. However, our diets were formulated to be isoenergetic and supply more than 2.7 Mcal ME/kg DM, including soybean oil in urea containing diets in order to compensate the reduction in the fat content caused by the replacement of soybean meal.

### 4.2. Ruminal Fermentation, Acid-Base Status and Biochemical Profile

Ruminal concentration of both ammonia-N and VFA and molar proportions of the individual VFA were unaffected by dietary treatments, suggesting that urea supplementation caused no changes in ruminal fermentation. Obviously, caution must be taken when interpreting data from only one sampling time [36], but the results of the in vitro assay showed a similar pattern regarding the effect of diet to those obtained in vivo. In addition, the lack of effect on feed intake and feed efficiency would be also in concordance with the lack of differences in the pattern of ruminal fermentation. Moreover, mean values were in line with those reported for fattening lambs fed rich-starch diets [37,38].

It must be pointed out that in the current trial diets were isonitrogenous and ammonia-N concentration in all diets was above the minimum required to maximize ruminal fermentation and microbial protein synthesis [39,40]. Ammonia-N concentration results from an equilibrium between absorption, utilization for microbial protein synthesis, and production from ruminal degradation of dietary or endogenous sources of N, so a greater ammonia-N production from dietary N is not necessary reflected in higher ruminal ammonia-N concentration. It has been reported that rate of ureagenesis determines the disposal of bicarbonate and contributes to the maintenance of pH homeostasis [41]. Therefore, the lower HCO_3_ and total CO_2_ concentrations in Urea2 lambs in comparison to lambs receiving the control diet could be due to a higher urea synthesis in the liver, as a consequence of a greater absorption of ammonia-N. Albumin and urea synthesis seems to be metabolically interconnected [42] and the higher plasmatic concentration of albumin in Urea2 lambs would be consistent with an increase in urea synthesis.

Values of urea plasma concentration were in line with those reported by Wang et al. [43] and they were unaffected by urea supplementation. Excess of dietary ammonia-N is the largest contributor to plasma urea, but a greater absorption of ammonia-N in lambs fed urea containing diets could be compensated by differences in the catabolism of dietary or endogenous protein [44]. Moreover, it is well known that urinary output of urea and ammonia-N is increased when degradable protein is given in excess [43,45], which would also compensate the increased ammonia-N absorption and urea synthesis, with minor fluctuations in plasma urea concentration.

The lower and greater concentration of HCO_3_ and albumin, respectively, from Urea2 lambs in comparison to lambs fed the control diet is in concordance with the lower blood pH recorded in the Urea2 lambs [41]. Therefore, it seems that urea supplementation could trigger mild metabolic acidosis, defined by a reduction in the buffer capacity accompanied with a slight reduction of blood pH within the physiological range. Despite of that, no adverse effects were observed either on hepatic cell integrity or lipid metabolism as suggested by the plasmatic levels of AST, ALT, cholesterol, and triglycerides. In a recent study comparing several levels of urea supplementation (0%, 0.5%, 1.5%, and 2.5%) in lambs, Wang et al. [43] reported effects of urea supplementation with higher levels of plasmatic concentrations of creatinine, triglycerides, and alkaline phosphatase when animals were fed diets with levels of urea over 1.5%. In our experiment, the maximum proportion of urea was 0.95% and additionally sodium bicarbonate was included in all experimental diets, which could have counteracted the possible effect of urea supplementation on blood pH.

In agreement with Noro et al. [46], glycaemia tended to increase with the proportion of urea in the diet. It has been suggested that this effect could be due to either an underutilization of glucose by insulin-sensitive extrahepatic tissues or an improvement of gluconeogenic capacity [47]. Neither plasmatic insulin concentration nor activity of enzymes involved in gluconeogenesis have been evaluated in the present study. However, urea supplementation did not affect the plasmatic concentrations of cholesterol and triglycerides, suggesting that insulin might not have been affected [48]. The lack of effect on postmortem muscle pH would also support this hypothesis.

### 4.3. Carcass and Meat Quality

Dressing percentage was similar to that reported by Eyal et al. [34] for male Assaf lambs of similar slaughter body weight but, as expected, greater than those reported by Rodríguez et al. [14] for male Assaf lambs slaughtered at 25 kg of BW. Likewise, proportions of prime and low grade commercial cuts (first and third categories, respectively) were lower and higher, respectively, than the values reported for lighter Assaf lambs [14]. Nevertheless, the effects of slaughter weight on carcass joint composition is variable across sheep breeds and ranges of slaughter weight [49,50] and, as far as we know, there are no published works comparing the carcass quality of light and heavy fattening Assaf lambs.

Meat chemical composition, cooking losses, lightness, and hardness were within the range of values reported by Cohen-Zinder et al. [51] for heavier Assaf male lambs. As expected meat showed greater values of fat content and yellowness and lower of lightness, redness, and tenderness than those reported for lighter fattening Assaf lambs [14]. Meat color and specially hardness were affected by storage time [52,53], and it was noteworthy that meat aging process was unaffected by diet.

Diet is the major extrinsic factor affecting carcass characteristics and hence the lack of effect of dietary treatments on carcass yield and characteristics would be in line with the observed results in feed intake and efficiency. Similar results have been reported by Rozanski et al. [16], who did not observe effects on muscle pH, dressing percentage, chilling losses and morphometric parameters in Dorper lambs fed diets with levels of urea from 0 to 1.5% of DM. These authors have suggested that urea supplementation could increase meat pH as consequence of a reduction of glycogen reserves in muscles. Nevertheless, as it has been previously discussed, the effect of urea supplementation on glucose metabolism is not clear and in our study meat pH was not affected by dietary urea supplementation.

Meat color and hardness are the most important factors influencing consumers’ choice and eating satisfaction. These parameters were not affected by urea supplementation, in line with the lack of differences between experimental groups in weight at slaughter or in meat pH and chemical composition and in concordance of the findings of de Carvalho et al. [54] and Wang et al. [15].

### 4.4. Feeding Costs

According to our results, partial replacement of 25% of soybean meal with urea would reduce the feeding cost per kg of CCW produced by approximately 7%, being lower the reduction observed with the highest level of urea (5%). Other studies have reported further improved benefits [55], but in the current trial the differential cost of the experimental diets was lowered by the inclusion of soybean oil. In any case, regular inclusion of feed grade urea in the diet of fattening lambs will reduce the demand of protein sources for sheep feeding, which can be used directly as food for humans. The use of urea as a feed ingredient might also reduce the carbon footprint and the environmental impact derived from soybean meal production and transport, especially when land use change is involved [56,57].

## 5. Conclusions

Replacement of soybean meal with feed grade urea up to 39% in protein-rich diets (16–17% CP) can support high growth rates in heavy fattening Assaf lambs (from 29 to 50 kg of BW) and reduce feeding costs, without adverse effects on animal feed efficiency and carcass or meat quality. Nevertheless, urea supplementation at levels of 1% of DM may trigger a mild metabolic acidosis that could affect animal health in the long term.

## Figures and Tables

**Table 1:** Ingredients and chemical composition of experimental diets.

	Control	Urea1	Urea2
**Ingredients (g/kg)**			
Barley	440	470	491
Corn	180	189	189
Soybean meal	190	142	115
Barley straw	150	150	150
Molasses	10	10	10
Urea	-	6	9.5
Soybean oil	-	3	6
Mineral-vitamin premix	25	25	25
Bicarbonate	5	5	5
**Chemical composition**			
Dry matter (DM; g/kg)	889	895	897
Neutral detergent fiber (g/kg DM)	193	201	200
Crude protein (g/kg DM)	160	156	163
Fat (g/kg DM)	22	20	24
Ash (g/kg DM)	68	68	67
Cost (€/kg DM)	0.387	0.377	0.372

**Table 2:** Feed intake, average daily gain, and feed efficiency of heavy lambs fed diets where soybean meal was partially replaced by urea [0% (Control), 25% (Urea1), and 39% (Urea2) of replacement].

	Control	Urea1	Urea2	SED ^1^	*p* Value
Dry matter intake, g/day	1514	1399	1457	73.7	0.309
Average daily weight gain, g/day	312	310	318	18.9	0.912
Feed conversion ratio, g/g	4.91	4.55	4.61	0.194	0.149
Residual feed intake, g	36.6	−34.7	4.0	35.21	0.151
Final body weight, kg	50.5	48.8	49.3	1.89	0.666

^1^ SED: Standard error of the difference.

**Table 3:** pH and volatile fatty acids (VFA) and ammonia-N concentration in the rumen fluid and in vitro fermentation parameters (gas and VFA production and ammonia-N and methane concentration) of heavy lambs fed diets where soybean meal was partially replaced by urea [0% (Control), 25% (Urea1), and 39% (Urea2) of replacement].

	Control	Urea1	Urea2	SED ^1^	*p* Value
**In vivo parameters**					
pH	6.69	7.05	6.77	0.209	0.318
Ammonia-N, mg/L	129	154	120	27.4	0.474
VFA concentration, mmol/L	67.5	40.5	42.6	13.84	0.142
Acetate, %	56.0	61.6	62.2	3.48	0.201
Propionate, %	32.0	22.8	25.3	4.09	0.108
Butyrate, %	5.59	7.34	6.23	1.023	0.248
Others, %	6.38	8.31	6.27	1.325	0.266
Acetate/Propionate	1.82	2.80	2.58	0.456	0.119
**In vitro fermentation**					
pH	6.74	6.69	6.75	0.204	0.945
Gas production, mmol	2.17	2.11	2.15	0.093	0.775
Methane, mmol	0.092	0.131	0.090	0.022	0.281
Ammonia-N, mg/L	352	405	393	49.4	0.557
VFA production, mmol	2801 ^b^	2553 ^a^	2752 ^b^	62.9	0.008
Acetate, %	50.8	53.3	48.6	2.38	0.204
Propionate, %	35.3	30.8	37.8	3.48	0.182
Butyrate, %	6.83	10.24	7.51	2.752	0.456
Others, %	7.10	5.68	6.05	1.472	0.621
Acetate/Propionate	1.44	1.85	1.30	0.275	0.166

^1^ SED: Standard error of the difference; ^a,b^ means with different superscript are significantly different (*p* < 0.05).

**Table 4:** Blood acid-base status of heavy lambs fed diets where soybean meal was partially replaced by urea [0% (Control), 25% (Urea1), and 39% (Urea2) of replacement].

	Dietary Treatments			SED ^1^	Sampling Days		SED ^2^	*p* Values		
	Control	Urea1	Urea2		Day 35	Day 63		Diet	Day	Diet*Day
pH	7.44	7.43	7.40	0.014	7.44	7.41	0.010	0.065	0.005	0.660
HCO_3_−, mmol/L	26.0 ^b^	25.5 ^ab^	24.5 ^a^	0.45	26.0	24.6	0.37	0.005	0.0004	0.184
pCO_2_^3^, mmHg	41.2	42.1	42.2	1.75	41.6	42.1	1.36	0.814	0.708	0.418
Anion_gap, mmol/L	12.0	12.3	13.1	0.55	12.1	12.9	0.29	0.187	0.017	0.513
tCO_2_^3^, mmol/L	27.2 ^b^	26.8 ^ab^	25.7 ^a^	0.49	27.3	25.9	0.40	0.011	0.001	0.185
Na, mmol/L	144.6	144.8	143.5	0.684	144.7	143.9	0.511	0.130	0.120	0.435
K, mmol/K	5.36	5.27	5.53	0.196	5.56	5.21	0.092	0.389	0.001	0.655
Cl, mmol/L	112.0	112.2	112.1	0.39	112.3	111.8	0.18	0.890	0.009	0.833

^1^ SED: Standard error of the difference to compare dietary treatments. ^2^ SED: Standard error of the difference to compare days; ^a,b^ means with different superscript are significantly different (*p* < 0.05). ^3^ pCO_2_: CO_2_ pressure; tCO_2_: total CO_2_.

**Table 5:** Biochemical profile of heavy lambs fed diets where soybean meal was partially replaced by urea [0% (Control), 25% (Urea1), and 39% (Urea2) of replacement].

	Dietary Treatments			SED ^1^	Sampling Days		SED ^2^	*p* Values		
	Control	Urea1	Urea2		Day 35	Day 63		Diet	Day	Diet*Day
Urea, mg/dL	42.4	45.0	40.8	1.92	36.0	49.4	1.53	0.105	0.001	0.102
Protein, g/L	59.1	59.0	59.9	1.04	61.2	57.5	0.85	0.616	0.001	0.074
Albumin, g/L	34.9 ^a^	37.3 ^b^	37.4 ^b^	0.94	36.6	36.5	0.68	0.027	0.941	0.053
ALT ^3^, U/L	17.8	19.7	16.4	2.86	16.4	19.5	2.34	0.512	0.193	0.594
AST ^3^, U/L	99.2	99.5	92.7	7.52	93.3	100.9	10.59	0.584	0.212	0.660
Creatinine, mg/dL	1.00 ^a^	1.09 ^b^	1.08 ^b^	0.022	1.01	1.11	0.017	0.001	0.001	0.085
Glucose, mg/dL	98.6	102.5	104.7	2.62	103.4	100.5	2.06	0.076	0.173	0.251
Ca, mg/dL	11.7	11.9	12.0	0.18	12.2	11.5	0.13	0.092	0.001	0.013
P, mg/dL	7.69 ^a^	8.53 ^b^	8.18 ^ab^	0.245	8.70	7.56	0.197	0.004	0.001	0.476
Cholesterol, mg/dL	62.3	62.8	68.8	3.41	61.1	68.2	1.97	0.115	0.013	0.470
Triglycerides, mmol/L	26.1	28.3	29.2	1.92	31.7	24.0	1.57	0.262	0.001	0.087

^1^ SED: Standard error of the difference to compare dietary treatments. ^2^ SED: Standard error of the difference to compare days; ^a,b^ means with different superscript are significantly different (*p* < 0.05). ^3^ ALT: Alanine aminotransferase; AST: Aspartate aminotransferase.

**Table 6:** Carcass parameters of heavy lambs fed diets where soybean meal was partially replaced by urea [0% (Control), 25% (Urea1), and 39% (Urea2) of replacement].

	Control	Urea1	Urea2	SED ^1^	*p* Value
Cold carcass weight, kg	24.7	24.0	24.1	1.13	0.493
Dressing percentage, %	48.9	49.2	49.2	0.96	0.950
Chilling losses, %	2.16	2.32	2.36	0.241	0.696
pH 24 h	5.81	5.81	5.80	0.055	0.959
Pelvic and renal fat, %	3.17	2.47	2.62	0.567	0.439
Proportion of cuts ^2^, %					
Higher priced joints	52.1	52.4	52.6	0.60	0.653
Medium priced joints	17.7	18.5	17.6	0.48	0.148
Lower priced joints	30.2	29.1	29.7	0.84	0.402
**Subcutaneous fat color**					
L*	62.6	63.3	62.8	2.13	0.955
a*	2.60	3.36	2.91	0.625	0.476
b*	10.2	10.6	9.5	0.89	0.424
**Leg tissue composition, %**					
Muscle	60.6	59.0	59.0	1.40	0.428
Fat	17.4	18.9	18.0	1.26	0.507
Bone	20.1	19.7	20.6	0.78	0.519
Others	1.91	2.47	2.37	0.496	0.492

^1^ SED: Standard error of the difference. ^2^ Higher (legs, loin, and fore ribs), medium (shoulders), and lower (breast, neck, and tail) priced joints.

**Table 7:** Chemical composition, cooking losses, shear force, and color of meat of heavy lambs fed diets where soybean meal was partially replaced by urea [0% (Control), 25% (Urea1), and 39% (Urea2) of replacement].

	Dietary Treatments			SED ^1^	Day		SED ^2^	*p* Value		
	Control	Urea1	Urea2		1	6		Diet	Day	Diet*Day
Chemical composition (g/kg)										
Water	75.80	75.12	75.26	0.334	-	-	-	0.122	-	-
Crude protein	19.87	20.44	20.24	0.319	-	-	-	0.216	-	-
Crude fat	2.67	2.94	2.92	0.269	-	-	-	0.563	-	-
Ash	1.09	1.12	1.09	0.023	-	-	-	0.477	-	-
Cooking losses, %	30.26	30.14	29.38	1.080	29.74	30.12	0.882	0.675	0.668	0.750
WBSF ^3^, N	72.87	67.95	76.53	7.008	80.15	64.74	5.722	0.474	0.009	0.783
L*	40.77	39.66	40.57	0.703	40.23	40.44	0.572	0.244	0.709	0.962
a*	7.67	7.89	7.88	0.320	7.26	8.36	0.261	0.748	0.001	0.698
b*	8.88	8.42	9.07	0.436	8.27	9.31	0.356	0.313	0.005	0.869
Hue angle	49.05	46.64	48.88	1.542	48.58	47.80	1.259	0.226	0.539	0.871
Chroma	11.79	11.58	12.06	0.443	11.08	12.54	0.362	0.557	0.001	0.781

^1^ SED: Standard error of the difference to compare dietary treatments. ^2^ SED: Standard error of the difference to compare days. ^3^ WBSF: Warner-Bratzler shear force.
